# Novel Vitamin D Receptor Mutations in Hereditary Vitamin D Resistant Rickets in Chinese

**DOI:** 10.1371/journal.pone.0138152

**Published:** 2015-09-30

**Authors:** Lee-Moay Lim, Xuan Zhao, Mei-Chyn Chao, Jer-Ming Chang, Wei-Chiao Chang, Hung-Ying Kao, Daw-Yang Hwang, Hung-Chun Chen

**Affiliations:** 1 Division of Nephrology, Department of Medicine, Kaohsiung Medical University Hospital, Kaohsiung, Taiwan; 2 Department of Biochemistry, School of Medicine, Case Western Reserve University, Cleveland, OH, United States of America; 3 Division of Genetics, Endocrinology and Metabolism, Department of Pediatrics, Kaohsiung Medical University Hospital, Kaohsiung, Taiwan; 4 Department of Medicine, Kaohsiung Municipal Hsiao-Kang Hospital, Kaohsiung, Taiwan; 5 Department of Clinical Pharmacy, School of Pharmacy Taipei Medical University, Taipei, Taiwan; 6 Master Program for Clinical Pharmacogenomics and Pharmacoproteomics, School of Pharmacy, Taipei Medical University, Taipei, Taiwan; 7 Center for Lipid and Glycomedicine Research, Kaohsiung Medical University Hospital, Kaohsiung Medical University, Kaohsiung, Taiwan; 8 Faculty of Renal Care, College of Medicine, Kaohsiung Medical University, Kaohsiung, Taiwan; University of Alabama at Birmingham, UNITED STATES

## Abstract

Hereditary 1, 25-dihydroxyvitamin D-resistant rickets (HVDRR), a rare recessive disease, is caused by mutation in the *VDR* gene encoding the vitamin D receptor leading to the resistance to vitamin D. We described a female toddler with initial presentation of leg tenderness and clinical features of HVDRR including severe rickets, hypocalcemia and hypophosphatemia without alopecia. Genetic analysis revealed novel compound heterozygous mutations of p.M4I and p.H229Q in patient’s *VDR* gene. In *cis* p.M4I with FOKI-F eliminated both translation start sites of the VDR protein. The p.H229Q VDR exhibited significantly reduced VDR transactivation activity with intact dimerization with RXR. Our report expanded the mutation spectrum of HVDRR, and provided the first case of a benign variant p.M4I plus a common p.M1T polymorphism leading to a pathogenic allele.

## Introduction

Hereditary 1,25-dihydroxyvitamin D-resistant rickets (HVDRR, OMIM 277440), also known as vitamin D-dependent rickets type II, is a rare autosomal recessive form of vitamin D resistance due to loss of functional vitamin D receptor (VDR)[1]. The VDR is expressed in most tissues of the body, including intestine, kidney, bone, and keratinocyte of hair follicles [2, 3]. With the recruitment of co-activators and co-repressors, ligand-activated VDR-RXR complex regulate the expression of multiple target genes in tissue expressing VDR[3]. In HVDRR, the VDR is defective due to mutations in *VDR* gene, leading to accumulation of high levels of 1,25(OH)_2_D_3_ concentration_._ Conversely_,_ Vitamin D-dependent rickets type I which is caused by 1α-hydroxylase deficiency has low level of 1,25(OH)_2_D_3_. Clinical features of HVDRR include rickets, hypocalcemia, secondary hyperparathyroidism, hypophosphatemia, elevated alkaline phosphatase, with or without alopecia. Children with HVDRR are resistant to all forms of vitamin D therapy and require intravenous calcium treatment. Alopecia due to the defective VDR activity within keratinocytes, appears in approximately two-thirds of cases and is considered a marker of disease severity [2, 4]. Nonsense mutations, missense mutations in the VDR DNA binding domain, and mutations affecting the VDR-RXR dimerization cause alopecia, while mutations affecting the ligand-mediated transactivation and co-activator recruitment do not affect hair growth[4].

Several polymorphisms in the VDR gene have been identified with possible pathological significance in osteoporosis, osteoarthritis, diabetes, cancer, cardiovascular disease and bone marrow density [5, 6]. Fok I polymorphism (rs2228570) is located in the exon 2 of the *VDR* gene with T to C polymorphism converting the first translation initiation codon from ATG to ACG. In human, two VDR proteins composed of 427 amino acids (Fok I-f allel) or 424 amino acids (Fok I-F allele) in length can be found that are distinguished by differential initiation codons.

In this study, we described a HVDRR patient without alopecia and identified two novel mutations in the *VDR* gene, one that alters the *VDR* mRNA translation initiation site and one that affects vitamin D binding activity.

## Material and Methods

### Identification of novel mutations in patients with HVDRR

The control subjects were obtained from Kaohsiung Medical University Hospital (KMUH) nephrology cohort. Informed consent in written form was obtained from the parent of investigated individual for publication of the case details and medical images. Genomic DNA was extracted from peripheral blood leukocytes. Exons 2 to 9 and intron-exon boundaries of the *VDR* gene were amplified by PCR and directly sequenced. PCR amplification primers were available upon request (Table 1). The reference sequences of *VDR* gene and mRNA were NG_008731.1 and NM_000376.2, respectively.

**Table 1:** Exon-flanking primers used for PCR in the human VDR *gene*.

Exons		Length (bp)
2F	AGCTGGCCCTGGCACTGACT	335
2R	GCTGTGAGCGCCGCATGTTC	
3/4F	TGGAGACCAGGGGGCCCAGA	364
3/4R	TGAGGCCCTGGCCCCAGATG	
5F	TCCTGGAGGAGCTGCTGGCC	629
5R	GGACTGAAGTCCTGCTTACCTGAAGAG	
6/7F	CCAGAGGGAAGCCTGGGGCT	417
6/7R	GTGGTGGATGAGTGATCTCCAACCC	
8F	GTGTGGCTTGAAGGCGTTTACTGGT	579
8R	TACTCCCCGCTCCCCAGGTC	
9F	CACTGGAGGGCTTTGGGGCC	513
9R	CTGCTGAGTAGCCGCCAGCC	

The study protocol was approved by the Institutional Review Board of the Kaohsiung Medical University Hospital (KMUH-IRB-980010). All clinical investigation was conducted according to the principles expressed in the Declaration of Helsinki.

### Subcloning and construction of VDR plasmids

Total RNA was collected from the peripheral blood leucocytes of the patient by standard Trizol method. The full-length H229Q VDR cDNA was obtained by reverse transcription of total RNA using the M-MLV RT-PCR kit with primers 5'-CCGGCCGGACCAGAAGCCTT-3' and 5'-CTGCTGAGTAGCCGCCAGCC-3' followed by subcloning into the pcDNA3.1/V5-His TOPO TA Expression Kit (Invitrogen). The wild-type (WT) VDR expression plasmid was generated by using the H229Q plasmid as a template with Quickchange II XL site-directed mutagenesis kit (Stratagene, La Jolla, CA) and single primer (5'-CTCTCCATGCTGCCCCACCTGGCTGACCTGGTCAG-3') method[7]. All plasmids were completely sequenced for correctness.

### Cell culture, transient transfection, Western blot, and coimmunoprecipitation (CoIP)

COS-7 and HEK293 cells were obtained from American Type Culture Collection (Manassas, VA). Expression vectors of the WT or H229Q VDR were co-transfected with the reporter plasmid D3R-Luc using FuGENE6 (Roche, USA) according to the manufacture’s protocol[2]. Cells were harvested 24 h after transfection, and total protein was extracted followed by Western blot analysis. For CoIP analysis, rabbit anti-HA (sc-805, Santa Cruz Biotechnology, Santa Cruz, CA) and anti-FLAG (F3165, St. St. Louis, MO) polyclonal antibody were used in the Western blot and co-immunoprecipitation analysis according to our published protocol [8].

### VDR transactivation assays

COS-7 and HEK293 cells were grown in DMEM containing 10% bovine serum in 6-well tissue culture plates. Cells were transfected in triplicate with 100 ng/well WT or mutant VDR expression plasmids and 125 ng/well VDRE-luciferase plasmid using FuGene HD transfection reagent (Promega, Madison, WI). Total protein concentration and VDR expression level were served as an internal control for transfection. Twenty-four hour after transfection, cells were incubated in DMEM containing 1% bovine growth serum with vehicle (ethanol) or 1,25(OH)_2_D_3_ followed by an additional incubation for 24 h. The cells were then lysed in 250 μl of lysis buffer and assayed for luciferase activity using the Luciferase Assay Kit (Promega, Madison, WI) with a FLx 800 fluorescence microplate reader (Biotek Instruments, Wincoski, UT).

### Statistical analysis

Results are shown as means ± SE. One-way ANOVA, followed by Tukey’s honestly significant difference test, was used to calculate statistical analyses. A *p* value of 0.05 was considered significant.

## Results

### Clinical findings

**Fig 1:**
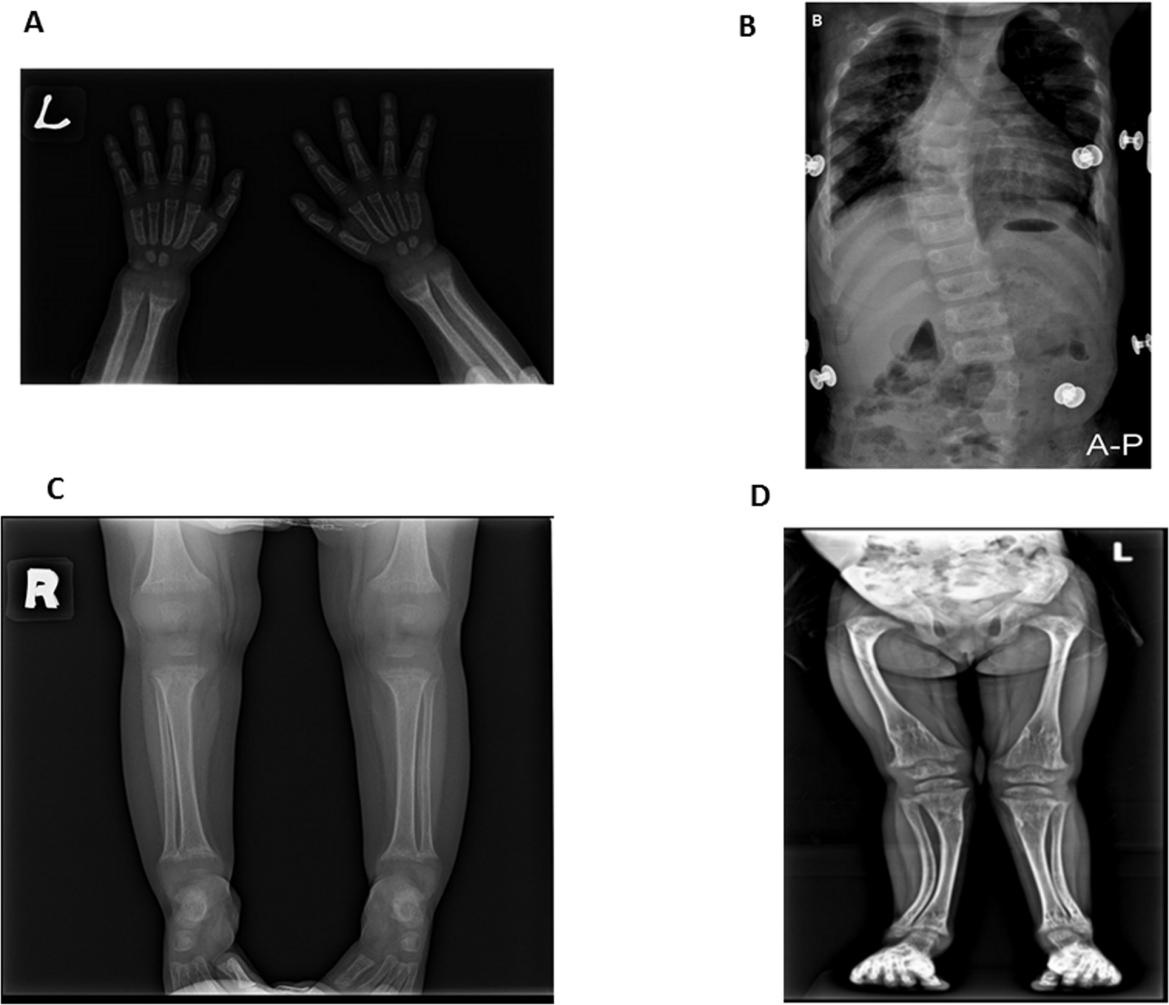
(A) Antero-posterior radiograph of the patient’s hand with rickets demonstrates cupping and fraying of the metaphyseal region. (B) Antero-posterior radiograph of the patient’s chest showing scoliosis of the thoracolumbar spine with Cobb’s angle of 31degrees. (C) Antero-posterior radiograph of lower extremities revealed widening between the distance of the end shaft and epiphyses. (D) The curvatures of legs revealed genu valgum with intermalleolar distance (IMD) of 5.0cm.

A 25-month old Han Chinese girl with body weight of 8.5 kg (<3^rd^ percentile, Z-score -4.5), body length of 70 cm (< 3^rd^ percentile, Z-score -3.9) initially presented with bilateral leg muscle weakness, bone pain, and growth delayed. She was able to sit but unable to stand or walk without support due to bone pain. The lateral and posterior fontanels were closed, but the cranial sutures and distribution of scalp hair were normal. Her serum chemistry panel revealed several abnormalities with total calcium (Ca) 7.2 mg/dL (8.4–10.2mg/dL), phosphorus 2.1 mg/dL (2.5–4.6 mg/dL), intact parathyroid hormone 586.4 pg/ml (11–62 pg/ml) and alkaline phosphatase 2038 U/L (32–92 IU/L)(Table 2). There was no seizure-like incident reported. Initially, she received daily treatment of 250 mg calcium carbonate and 0.25 μg of Calcitriol. At 3 years of age, she was referred to us for further examination and showed 25(OH)D of 17.3 ng/ml (30–74 ng/ml) and 1,25(OH)_2_D_3_ of 285 pg/ml (20–45pg/ml). The skeletal examination revealed generalized osteopenia with cupping and fraying at the metaphyseal ends of long bones of upper and lower extremities, with widening of growth plates and epiphyses (Fig 1A and 1C). Severe osteopenia leads to spine scoliosis and compression fracture of the 8^th^ thoracic spine (Fig 1B). The curvatures of legs revealed genu valgum with intermalleolar distance (IMD) of 5.0cm (Fig 1D). There was no rachitic rosary observed. There was no evidence of nephrocalcinosis based on abdominal sonography examination. Complete or partial alopecia was not found. Hereditary vitamin D resistant rickets was suspected and patient received oral 1000 mg calcium carbonate and 1 μg of Calcitriol treatment daily. Despite increased oral calcium and Calcitriol supplement, the serum calcium level and the clinical symptoms did not improve after 6 months of treatment. At the age of 5, when her serum iPTH elevated to 715.71 pg/ml, intravenous calcium infusion was given after the course of inpatient hospitalization with the implantation of intravenous catheter. After the infusion, her serum iPTH level was reduced to 430.15 pg/ml and serum calcium of 9.3 mg/dL before she was discharged. She maintained weekly calcium infusion (108.8 mg) plus oral calcium and calcitriol at outpatient clinic. Two episodes of pneumonia occurred after catheter implantation, which were successfully treated.

**Table 2:** Patient’s biochemical profile.

	Initial values	During Calcium infusion	Follow up
**Calcium, mg/dL (8.4–10.2)**	7.2	7.4	9.3
**iCa, mg/dL (4.64–5.28)**	4.18	3.53	5.49
**Phosphorus, mg/dL (2.5–4.6)**	2.1	2.9	3.1
**Alkaline phosphatase, U/L (32–92)**	2038	1057	1266
**iPTH, pg/ml (11–62)**	586.4	715.7	430.1
**25(OH)D, ng/ml (30–74)**	17.3		
**1,25(OH)**_**2**_ **D**_**3**_ **, pg/ml (20–45)**	285*		

* This data was collected during oral calcium and active Vitamin D supplement.

### Identification of novel VDR mutations

**Fig 2:**
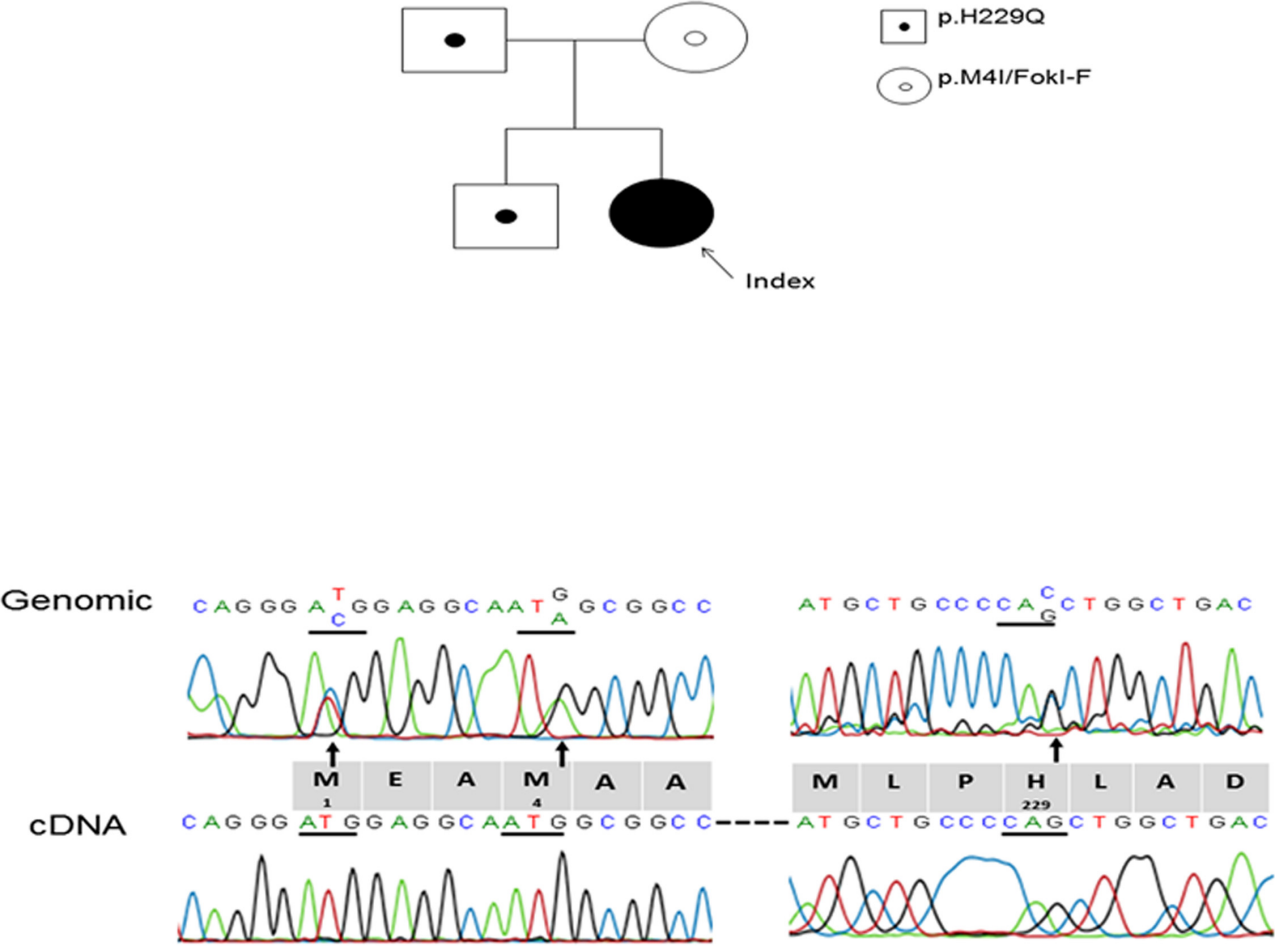
Family pedigree and DNA sequences. (A) Family pedigree showing *VDR* mutation status. Small solid circle indicates p.H229Q carrier, while small open circle indicates p.M4I/FOKI-F carrier. Index case is large solid circle (B) Heterozygous variants in c. 2T>C (p.M1T), c. 12G>A (p.M4I), and c.687C>G (p.H229Q) were found in patient’s genomic DNA. Full-length cDNA derived from patient’s peripheral lymphocyte showed amino acids M1, M4, and H229Q are located on the same allele.

To elucidate the genetic components of this phenotype, Sanger sequencing was used to identify mutations in the *VDR* gene and the data indicated mutations at c.12G>A (p.M4I), c.687C>G (p.H229Q), and heterozygous f/F of the *Fok I* polymorphism (rs2228570) (Fig 2B, upper panel). Further cDNA analysis of this patient and her family segregation analysis showed the F and c.12G>A alleles (F/M4I) were *in-cis* and derived from her mother while f and c.687C>G (f/H229Q) were on the same allele inherited from the father (Fig 2A). Her parents are clinically asymptomatic with normal serum calcium, phosphate, and iPTH level. In the patient’s cDNA analysis, only the f/H229Q full-length cDNA was sub-cloned from patient’s peripheral blood while no full-length F/M4I cDNA can be identified among 20 isolated subclones.

**Fig 3:**
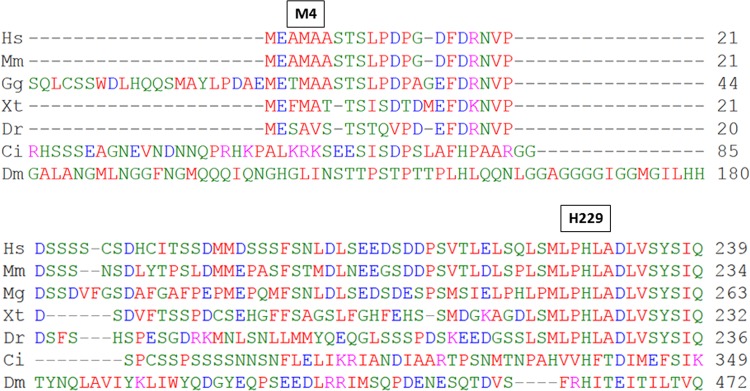
The conservation of amino acid H229 of *VDR* gene in different species. Clustal Omega (http://www.ebi.ac.uk/Tools/msa/clustalo/) was used for sequence alignment of VDR from different species. Hs, *Homo sapiens*, Mm, *Mus musculus*, Gg, *Gallus gallus*, Xt, *Xenopus tropicalis*, Dr, *Denio rario*, Dm, *Drosophila melanogaster*, Ci, *Ciona intestinalis*.

Neither c.12G>A (p.M4I) nor c.687C>G (p.H229Q) variants was existed in the 1000 Genomes Projects (http://browser.1000genomes.org/index.html), Exome Variant Server of NHLBI Exome Sequencing Project (http://evs.gs.washington.edu/EVS/), NCBI SNP database (http://www.ncbi.nlm.nih.gov/projects/SNP/), or HGMD (http://www.hgmd.cf.ac.uk/ac/index.php). Furthermore, both variants were not found in 100 healthy control Han Chinese examined by restriction fragment length polymorphism method analyzed by BsrdI or PuvII digestion (data not shown). The amino acid at position H229 is well conserved from human to fruit fly while M4 is only conserved (Fig 3) to frog. The prediction software Polyphen-2 and MutatonTaster suggested probable damage (HumVar score: 0.954) and disease-causing for p.H229Q mutation, and benign (HumVar score: 0.002) and tolerated for p.M4I mutation.

### Functional studies of p.H229Q mutant

**Fig 4:**
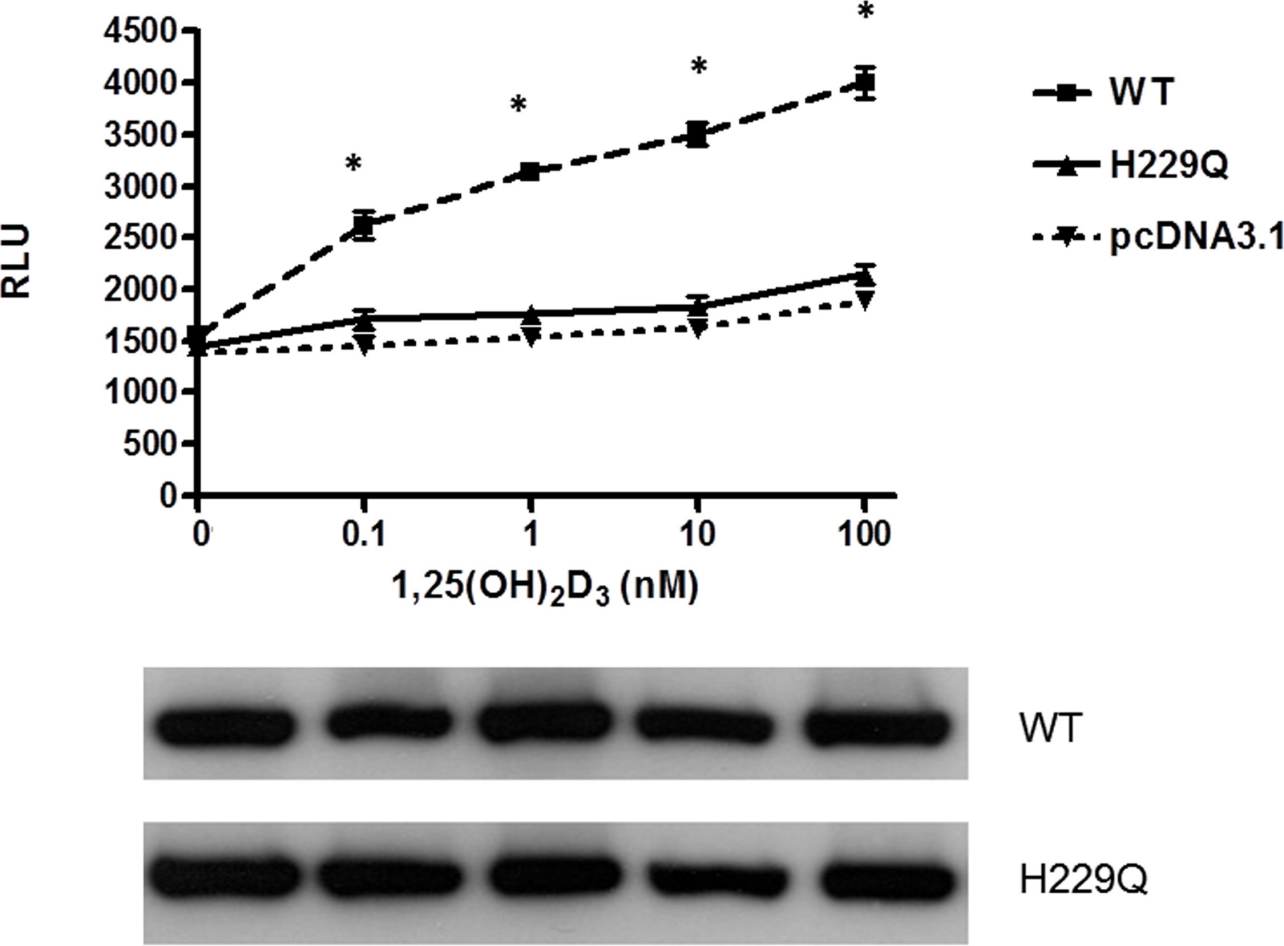
The VDR transactivation activity is markedly reduced in p.H229Q mutant. COS-7 cells were transfected with pcDNA3.1 empty vector, pcDNA3.1-WT (WT) or pcDNA3.1-H229Q (H229Q) mutant VDR expression vectors and a VDRE-luciferase reporter. Cells were treated with different concentrations of 1,25(OH)_2_D_3_ for 24 h and luciferase activity measured. VDR protein levels in transfected cells at the five different 1,25(OH)_2_D_3_ concentration are shown. Results represent the average of at least 3 independent experiments performed in triplicates and are annotated as means±SE. *p<0.05 compared with control tested using one-way ANOVA.

**Fig 5:**
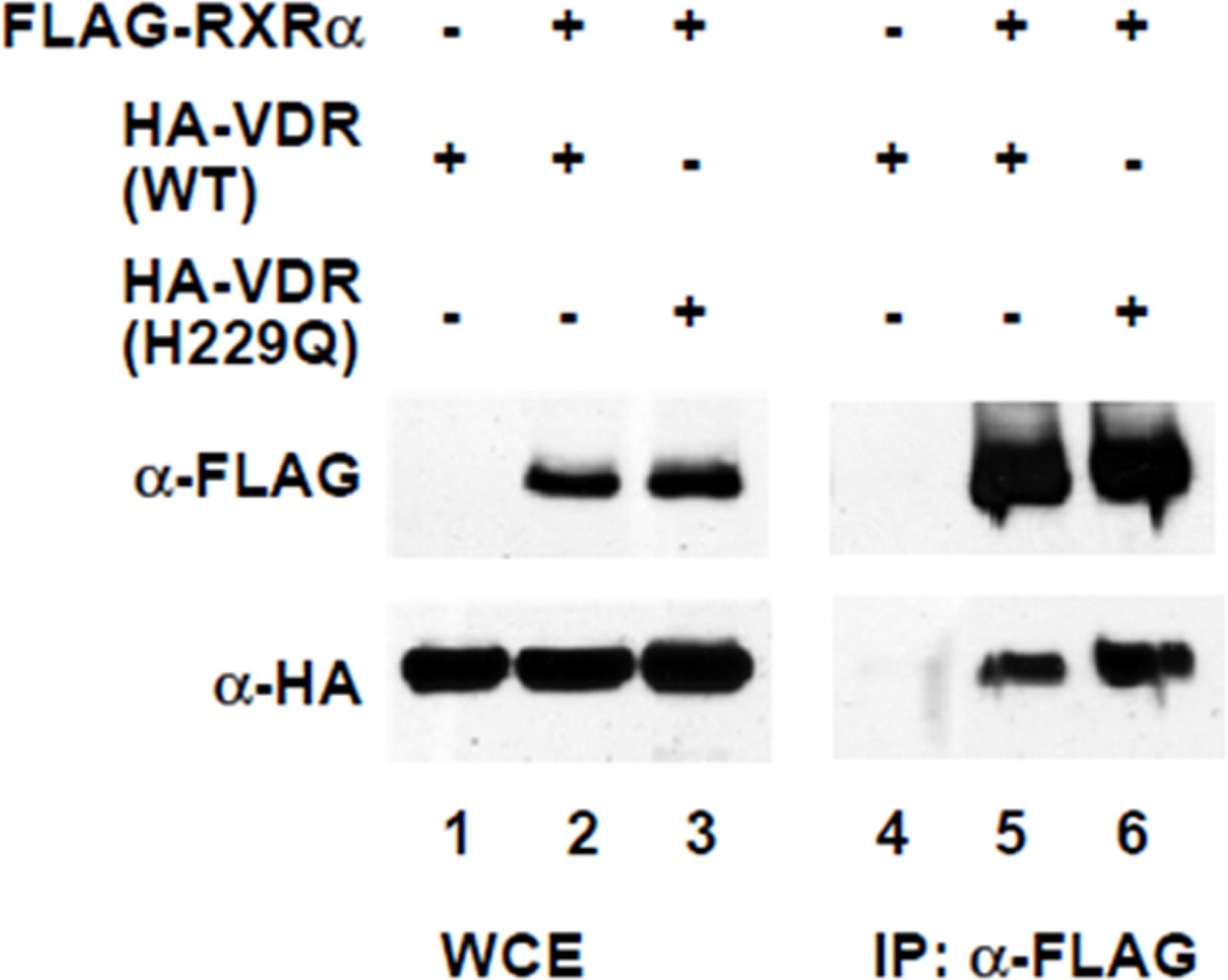
Heterodimerization of wild-type and mutant p.H229Q VDR with RXR. HEK293 cells were transfected with wild-type or p.H229Q mutant HA-VDR with FLAG-RAR expression plasmid. Cells were harvested, extracts prepared and coimmunoprecipitated with anti-FLAG antibody. The immunopellets were subjected to Western blotting with anti-HA and anti-FLAG antibodies. WCE, whole cell extract.

To determine whether p.H229Q mutant affect VDR transcriptional activity, we carried out transient transfection experiments using a reporter construct harboring a vitamin D response element. WT VDR exhibited a dose-dependent increase in luciferase activity in response to 1,25(OH)_2_D_3_ treatment, whereas the reporter activity of p.H229Q mutant was markedly reduced even up to 100 nM of 1,25(OH)_2_D_3_ (Fig 4). Immuno-blotting showed that both WT and p.H229Q VDR were expressed at similar protein levels (Fig 4). These results demonstrated that the p.H229Q mutation reduces the transactivation activity of the VDR. Furthermore, co-immunoprecipitation studies suggested that the p.H229Q VDR did not interfere with the heterodimerization of VDR and RXR (Fig 5).

## Discussion

HVDRR is a very rare form of rickets with ~100 cases reported worldwide. It is an autosomal recessive disease with males and females equally affected[9]. Affected children developed hypocalcemia within months of birth and followed by clinical rickets’ phenotype [1, 10]. Bone pain, muscle weakness, hypotonia of these patients are commonly observed, which lead to delays in walking ability. They are usually growth retarded, and in some cases the children developed severe dental caries and hypoplasia of the teeth [11, 12]. The children exhibit low serum concentration of calcium and phosphate and elevated PTH levels. Their serum 1,25(OH)_2_D levels are unusually high which is a major clinical observation that distinguishes HVDRR from 1α-hydroxylase deficiency[9]. Although most cases are recessive, a heterozygous missense mutation (E420A mutation), acting in a dominant-negative fashion over the wild-type VDR, which resulting in an attenuated response to 1,25(OH)_2_D_3_, was reported recently[10]. An unusual form of rickets, vitamin D-dependent rickets type 2B, was reported to have abnormal expression of a hormone response element-binding protein C1/C2 that interferes with the normal function of the vitamin D receptor [13, 14].

Currently there are more than 54 HVDRR-related *VDR* mutations reported in the literature and Human Gene Mutation Database (HGMD, http://www.hgmd.org). Mutations that are associated with 1,25(OH)_2_D resistance include mutations in DNA binding domain and ligand binding domain, nonsense mutations, insertions/substitutions, insertions/duplications, deletion and splice site mutations[9]. Our current studies extend mutation spectrum of VDR by studying two novel variants found in ethnic Han Chinese. The functional changes of p.M4I VDR (in terms of DNA binding, RXR heterodimerization, or co-activator/repressor binding) was unknown, while computational modeling predicted tolerated, polymorphism, and benign (SIFT, MutationTaster, and Polyphen-2, respectively). Thus, the p.M4I variant is probably a non-deleterious variant by itself; however, in the presence of F polymorphism, the first two translation initiation start sites of the VDR mRNA were abolished. The F/M4I mRNA expression was not found in the full-length VDR mRNA transcripts. Putatively, several aberrant translation start sites downstream may be used and generate either frame-shift terminations or N-terminal truncated VDRs.

The amino acid residue H229 is located in the H3 helix, which forms part of the ligand binding domain (LBD) important for the binding of 1,25(OH)_2_D_3_[15, 16]. Several previous publications showed H229A marked eliminated ligand binding, VDR-activated transcription, and heterodimerization with RXR in vitro[17–19]. H229 residue does not directly contact the ligand, but is critical for the agonistic conformation of the VDR by bringing the helix 3 and the β-sheet together with a hydrogen bond, thus stabilizing the receptor structure[19]. Replacing H229 with alanine leads to a labile receptor protein that is biologically inactive, possible due to lose of the hydrogen bonding with Y295 that connect the H3 helix and β-sheet[19]. Previously, there are several studies reported the roles of H229 of human vitamin D receptor but most of them are based on animal and cell studies[18, 19]. Our functional studies, which are the first in human, confirmed that H229Q impaired VDR-activated transcription but not heterodimerization with RXR. Furthermore, the lack of alopecia in our patient indicated that H229Q VDR do not interfere with the heterodimerization with RXR, since patients with mutations affecting the VDR-RXR heterodimerization (residues F251, Q259, V346, and R391) all presented with alopecia[3, 14, 20]. This conclusion was supported by our data indicating that mutant VDR and RXR still dimerize. Recently, a HVDDR individual with homozygous p.L227P mutation showed severe clinical symptoms without alopecia [21].

Hypocalcemia in HVDRR patient leads to secondary hyperparathyroidism and hypophosphatemia, causing a decrease in bone mineralization and the development of rickets. Some patients improved after treated with pharmacological doses of vitamin D ranging from 5000–40000IU per day, 20–200ug per day of 25(OH)D_3_, and 17–20ug per day of 1,25(OH)_2_D_3_ [1, 22]. When patients fail to response to the above regimens, intensive calcium therapy is used, including intravenous calcium infusion in children who failed prior treatments with high doses of vitamin D derivatives and/or oral calcium[20, 23]. Oral calcium can be absorbed in the intestine, while intravenous form of calcium infusion bypasses the calcium absorption defect in the intestine caused by lack of action of the mutant VDR[24]. Although patients without alopecia usually have milder clinical symptoms, this individual had severe rickets with osteoporosis and compression fracture. Enteral calcium infusion should be considered based on a previous successful treatment [21], since complication like infection and sepsis may recur during intravenous calcium infusion. In one report, cinacalcet adjunctive therapy with high doses of oral calcium effectively normalized the metabolic abnormalities and bone condition, which should be considered for this patient [25]. Recently, enteral calcium infusion was used successfully for a HVDRR patient [21].

The influence of vitamin D deficiency on various inflammatory processes has raised concern in addition to its well-established role in systemic calcium homeostasis[26]. 1,25(OH)_2_D_3_ influences adaptive immunity and inflammation through the regulation of T-cell helper 1(TH 1), TH2 production and IL-17[14]. Increasing evidences show that there is impaired innate and adaptive immune function in HDVRR patients [27]. However, little information on infection, inflammation and autoimmune disease was reported in these patients, suggesting that the underlying VDR-independent compensatory mechanism remains to be determined[5]. In addition to pneumonia, our patient also suffered from recurrent episodes of bacterial sinusitis with improvement after taking oral antibiotics, and whether these infections were associated with vitamin D related immunodeficiency remains unclear. At present, there was no autoimmune issue observed.

## Conclusion

In conclusion, we have extended the mutation spectrum of HVDRR by studying two novel VDR mutations. Our case study demonstrates another example that severe metabolic abnormalities can present without alopecia and contributions of Fok I and a benign p.M4I variant in a HVDRR patient.
